# Polymyalgia Rheumatica Post-SARS-CoV-2 Infection

**DOI:** 10.1155/2024/6662652

**Published:** 2024-03-14

**Authors:** Carolina Duarte-Salazar, José Eugenio Vazquez-Meraz, Lucio Ventura-Ríos, Cristina Hernández-Díaz, José Arellano-Galindo

**Affiliations:** ^1^Departmento de Reumatología, Instituto Nacional de Rehabilitación Luis Guillermo Ibarra Ibarra, Mexico City, Mexico; ^2^Departamento de Hematología, Hospital General de México Dr. Eduardo Liceaga, Mexico City, Mexico; ^3^Laboratorio de Virología Clínica y Experimental, Unidad de Investigación en Enfermedades Infecciosas, Hospital Infantil de México Federico Gómez, Mexico City, Mexico

## Abstract

There is growing evidence that infection with severe acute respiratory syndrome coronavirus 2 (SARS-CoV-2) can lead to dysregulation of the immune system and, consequently, the development of autoimmune phenomena. Here, we describe the case of a 75-year-old woman with rheumatic manifestations characterized by intense musculoskeletal pain and stiffness in the neck and shoulders, with sudden onset and with the inability to raise her arms. The patient was admitted with severe pain located in the neck and shoulders. Previously, she had oropharyngeal pain, severe fatigue, and fever; a real-time polymerase chain reaction test for COVID-19 was positive. Two weeks later, the patient presented localized musculoskeletal pain in the neck and shoulders. Relevant laboratory results included an erythrocyte sedimentation rate of 46 mm/hr and a negative rheumatoid factor test; ultrasound findings with bilateral subacromial–subdeltoid bursitis were observed. A diagnosis of polymyalgia rheumatica (PMR) was initially made according to the EULAR/ACR provisional classification criteria for PMR; however, due to C-reactive protein negativity, the diagnosis was established based on symptoms. Management was with prednisone at the dose of 25 mg/day for 4 weeks and progressive reduction until prednisone suspension. The patient showed complete recovery at 6 months of follow-up. In this case, COVID-19 was implicated in the development of autoimmune and inflammatory rheumatic manifestations. PMR is a rare rheumatic condition that should be included in the wide range of rheumatologic manifestations expressed post-SARS-CoV-2 infection.

## 1. Introduction

There is growing evidence that infection with SARS CoV2 can lead to dysregulation of the immune system and, consequently, the development of autoimmune phenomena. This immune dysregulation is due to the production of antibodies at the onset of autoimmune or inflammatory rheumatic diseases [1–3]. Numerous COVID-19 patients with autoimmune and rheumatic manifestations have been reported. In this context, we present a case of polymyalgia rheumatica (PMR) identified in a patient after being diagnosed with COVID-19 2 weeks earlier.

## 2. Case Presentation

**Figure 1:**
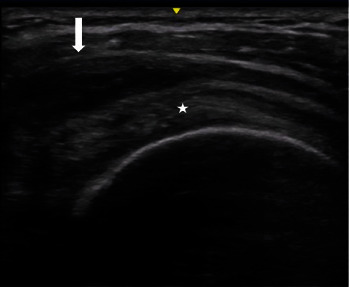
Subacromial–subdeltoid bursa distended with hypoechoic content compatible with bursitis (arrow). Supraspinatus tendon in cross-section (star).

**Figure 2:**
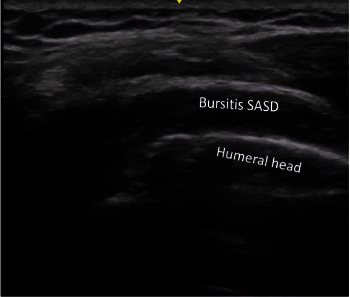
Subacromial–subdeltoid bursitis conditioned tendon impingement to the abduction movement.

Previously, our 75-year-old female patient was a healthy subject with no history of autoimmune or rheumatic disease. She noted in her medical history that she had received the Pfizer-BioNTech COVID-19 vaccine. Four months later, she had oropharyngeal pain, severe fatigue, and fever; a real-time polymerase chain reaction (PCR) test for COVID-19 was positive, and she was diagnosed with COVID-19. Two weeks later, the patient began to have severe arthralgias and musculoskeletal pain of sudden onset localized in the neck, shoulders, and forearms, morning joint stiffness that persisted throughout the day, and a 4 kg weight loss. On physical examination, she had no fever and no arthritis but limited ability to raise her arms. The remainder of the physical examination was ordinary. Laboratory tests revealed an erythrocyte sedimentation rate (ESR) of 46 mm/hr, a normal number of white blood cells, a normal concentration of C-reactive protein (CRP), and a negative result for rheumatoid factor (RF). Ultrasound findings with bilateral subacromial–subdeltoid bursitis were observed (Figures 1 and 2). The diagnosis of PMR was initially made using the criteria set by the European League Against Rheumatism and American College of Rheumatology (EULAR/ACR). However, since our patient had a negative CRP result, we relied on their reported symptoms to make the diagnosis [4, 5]. The patient was managed with prednisone at a dose of 25 mg/day for 4 weeks with immediate improvement and progressive prednisone reduction until suspension. The patient showed complete recovery at 6 months of follow-up. All diagnostic and treatment procedures were performed after obtaining informed consent and in accordance with the guidelines of the Helsinki Declaration.

## 3. Discussion

PMR is considered the most common inflammatory rheumatic disease occurring in older adults [3]. The typical presentation of PMR involves sudden onset and severe pain in both shoulders and pelvic girdle, with morning stiffness lasting more than 45 min. No specific laboratory tests are available. The diagnosis of PMR is typically linked to inflammatory markers such as ESR and/or CRP, which are essential markers for diagnosis under EULAR/ACR guidelines [5]. Despite the absence of a positive CRP result in our patient, we relied on the use of symptoms to establish the diagnosis, as has been done in previous cases [6]. SARS CoV-2 has been involved in PMR and even was observed to be associated with a relapse in a patient who was previously in remission [4]. A previous study found that a subset of patients diagnosed with PMR did not have a positive CRP result at diagnosis, with many experiencing prolonged symptoms and being younger [7]. While our patient also experienced symptoms for a significant period, she was not young. The available evidence shows that the COVID-19 cohort exhibited substantially higher risks of autoimmune diseases compared to non-COVID-19 individuals [8]. Cases of autoimmune diseases have been reported following severe acute respiratory syndrome coronavirus 2 (SARS-CoV-2) infection, such as systemic lupus erythematosus, antiphospholipid syndrome, immune thrombocytopenia, vasculitis, rheumatoid arthritis, seronegative spondyloarthropathy, Kawasaki disease, and myositis, and there is also evidence of large vessel vasculitis in so-called long COVID-19 [9].

Here, we report a clinical case of PMR in an older adult who had been diagnosed with COVID-19 2 weeks earlier. The diagnosis of PMR was made by the support the classification criteria of EULAR/ACR 2012 plus ultrasound findings [5]. In these criteria, a score of 5 or more is required in the ultrasound algorithm. The following criteria were met: morning stiffness duration >45 min (2 points), absence of RF and/or anticitrullinated protein antibody (ACPA) (2 points), absence of other joint involvement (1 point), and at least one shoulder with subdeltoid bursitis (1 point with ultrasound), with a total score of 6 points. Abnormal CRP and/or ESR are required; however, our patient was CRP negative; thus, we decided to support the diagnosis on symptoms [4, 6]. Recently, a study identified 62 patients among 454 patients affected with PMR with normal baseline values of ESR and CRP [10]. Patients with relapse of PMR and the proportion of glucocorticoid-free remission during the 24 months of follow-up were not significantly different in patients with normal baseline versus elevated baseline for ESR or CRP. Patients with relapse of PMR and the proportion of GC-free remission during 24 months follow-up were not significantly different in patients with baseline normal versus baseline elevated ESR or CRP [7]. However, recently, it was shown that patients with a positive PCR test result for COVID-19 had significantly higher risks of autoimmune diseases and mortality when compared to PCR test-negative non-COVID-19 controls [8].

Because the diagnosis is very challenging in an elderly patient with a bilateral painful shoulder associated with inflammatory biomarkers (CRP or ESR), exclusion of other disorders that mimic PMR is mandatory before making the diagnosis of PMR. Our patient did not have an autoimmune or rheumatic history, symptoms, or signs of inflammation in other joints. There are various causes of chronic subacromial–subdeltoid bursitis in elderly patients related to rheumatoid arthritis, seronegative arthritis, bursitis related to calcium pyrophosphate dihydrate crystal deposition disease, and/or osteoarthritis generating a nonspecific response leading to rice body formation [11, 12]. Rice bodies are the rare and unique sonographic presentation of chronic bursitis [13]. Nevertheless, our patient was suffering from acute nonchronic shoulder pain that was temporarily associated with post-COVID-19 infection, with ultrasound analysis showing a well-defined hypoechoic area. Notably, our patient, who had no history of rheumatic disease, had a close temporal association with SARS-CoV-2 infection and clinical manifestations of PMR.

Given that infections are environmental triggers of autoimmunity, an autoimmune response would also be expected in COVID-19 [14]. The three mechanisms that explain the development of autoimmunity are (i) molecular mimicry, when a pathogenic antigen shares structural similarities with self-antigens; (ii) spreading of epitopes, which facilitates the development of immune responses to endogenous epitopes secondary to the release of self-antigens during a chronic autoimmune or inflammatory response, the immune response to the pathogen, or the pathogen itself causing tissue lysis, after which the released (neo-) antigens are taken up by cells presenting antigen and cause a secondary immune response; and (iii) bystander activation, characterized by autoreactive B and T cells undergoing activation in an antigen-independent manner; nonspecific antigens are exposed through damaged tissue secondary to an inflammatory environment influencing the development of autoimmunity [14]. The definite mechanisms by which autoimmunity phenomena occur are not yet fully understood [15].

Recently, the multi-institutional US Collaborative Research Network (2023) [8] studied a retrospective cohort of participants with positive results of the PCR test for SARS-CoV-2, while the controls consisted of participants without COVID-19 (negative PCR tests and no diagnosis of COVID-19). The network estimated the risks of autoimmune diseases in both groups at 6 months of follow-up. Patients with a positive PCR test result for COVID-19 had a significantly higher risk of autoimmune diseases, exhibiting an adjusted hazard ratio for PMR of 2.90, 95% CI 2.36–3.57 [8].

## 4. Conclusion

It is important to document clinical cases of post-COVID-19 infection associated with autoimmune diseases because the SARS-CoV-2 virus is an environmental agent that strongly triggers autoimmunity. In the clinical setting, this raises concern about ending the monitoring of these patients before the development of autoimmunity. Evidence of autoimmune phenomena in these patients may also contribute to the development of post-COVID-19 syndrome. For patients with autoimmune diseases that occur after COVID-19, a prolonged follow-up time is necessary.

## Data Availability

Data are available upon request from the corresponding author.
